# Fetal outcome in emergency versus elective cesarean sections at Souissi Maternity Hospital, Rabat, Morocco

**DOI:** 10.11604/pamj.2016.23.197.7401

**Published:** 2016-04-15

**Authors:** Soukayna Benzouina, Mohamed El-mahdi Boubkraoui, Mustapha Mrabet, Naima Chahid, Aicha Kharbach, Amine El-hassani, Amina Barkat

**Affiliations:** 1 National Reference Center in Neonatology and Nutrition, Children's Hospital, University Hospital, Rabat, Morocco; 2 Research Team on Health and Nutrition of Mother and Child, Faculty of Medicine and Pharmacy of Rabat, Mohammed V University of Rabat, Morocco; 3 Departement of Public Health, Faculty of Medicine and Pharmacy of Rabat, Mohammed V University of Rabat, Morocco; 4 Souissi Maternity Hospital, Rabat, University Hospital, Morocco; 5 Cheikh Zaid Hospital, Abulcasis International University of Health Sciences, Rabat, Morocco

**Keywords:** Elective cesarean section, emergency cesarean section, fetal outcome

## Abstract

**Introduction:**

Perinatal mortality rates have come down in cesarean sections, but fetal morbidity is still high in comparison to vaginal delivery and the complications are more commonly seen in emergency than in elective cesarean sections. The objective of the study was to compare the fetal outcome and the indications in elective versus emergency cesarean section performed in a tertiary maternity hospital.

**Methods:**

This comparative cross-sectional prospective study of all the cases undergoing elective and emergency cesarean section for any indication at Souissi maternity hospital of Rabat, Morocco, was carried from January 1, to February 28, 2014. Data were analyzed with emphasis on fetal outcome and cesarean sections indications. Mothers who had definite antenatal complications that would adversely affect fetal outcome were excluded from the study.

**Results:**

There was 588 (17.83%) cesarean sections among 3297 births of which emergency cesarean section accounted for 446 (75.85%) and elective cesarean section for 142 cases (24.15%). Of the various factors analyzed in relation to the two types of cesarean sections, statistically significant associations were found between emergency cesarean section and younger mothers (P < 0.001), maternal illiteracy (P = 0.049), primiparity (P = 0.005), insufficient prenatal care (P < 0.001), referral from other institution for pregnancy complications or delivery (P < 0.001), cesarean section performed under general anesthesia (P < 0.001), lower birth weight (P < 0.016), neonatal morbidity and early mortality (P < 0.001), and admission in neonatal intensive care unit (P = 0.024). The commonest indication of emergency cesarean section was fetal distress (30.49%), while the most frequent indication in elective cesarean section was previous cesarean delivery (47.18%).

**Conclusion:**

The overall fetal complications rate was higher in emergency cesarean section than in elective cesarean section. Early recognition and referral of mothers who are likely to undergo cesarean section may reduce the incidence of emergency cesarean sections and thus decrease fetal complications.

## Introduction

Cesarean section delivery represents the most important operation in obstetrics and its incidence is on the rise throughout the world [1]. This increasing rate does not seem to improve the overall fetal outcome but is linked with increased morbidity and costs [2–4]. According to WHO, cesarean section rate greater than 15% is not justified in any region in the world [5, 6]. Cesarean section can be done as an elective as well as emergency procedure. This study was therefore undertaken to compare the fetal outcome and the indications in elective versus emergency cesarean sections in a tertiary maternity hospital.

## Methods

**Study design and location:** We conducted a comparative cross-sectional prospective study from January 1, to February 28, 2014 at Souissi maternity hospital of Rabat, Morocco.

**Inclusion and exclusion criteria:** All mothers undergoing cesarean section for any indication during the study period were included, except those who had definite antenatal complications that would adversely affect neonatal outcome.

**Data collection:** Data were prospectively collected for each birth by the attending pediatric resident: inpatient registration number, referral status, maternal socioeconomic status, maternal age and blood group, maternal weight and height, gravidity and parity, prenatal care (the number of prenatal visits and obstetric ultrasonography was retrieved), history of miscarriage or infertility, previous perinatal death, maternal chronic disease (chronic hypertension, diabetes mellitus), pregnancy complications (gestational hypertension, gestational diabetes mellitus, third trimester bleeding, premature rupture of membranes, pre-eclampsia, eclampsia, HELLP syndrome, placental abruption, placenta previa, intrapartum fever, documented urinary tract infection, prelabor rupture of membranes), presentation of the fetus, mode of delivery (elective or emergency cesarean section), indication of cesarean section, type of anesthesia (general anesthesia or regional block), date and time of birth, newborn's sex and weight, gestational age, Apgar score, abnormalities on physical examination and initial care to the newborn, need for resuscitation, newborn outcome until discharge, admission in neonatal intensive care unit. The following investigations were conducted in newborns to confirm suspected congenital malformations: thoracoabdominal radiograph, abdominal ultrasonography, transfontanellar ultrasonography, and echocardiography. No further follow up was done after discharge.

**Ethical considerations:** Informed consent was obtained from each participant. Ethical clearance was obtained from the ethics committee of the faculty of medicine and pharmacy of Rabat. The collection of data was made with the approval of the department head and was anonymous using the inpatient registration number.

**Definition of terms:** Cesarean section delivery is defined as the birth of a fetus, living or dead through an incision on the abdominal and uterine wall. The removal of the fetus from the abdominal cavity as in case of either ruptured uterus or abdominal ectopic pregnancy is excluded. Cesarean section delivery was classified as elective if the decision to perform the operation was made before the onset of labor and after preoperative preparation at a prearranged time during office hours to ensure the best quality of obstetrics, anesthetic, neonatal, and nursing services even when labor started before the operation (regular contractions with cervical dilatation). All others were considered as emergency cesarean deliveries. Parity was the number of previous pregnancies ending after 20 completed weeks of gestation including stillbirth. A woman was considered to have received adequate prenatal care when she had 3 or more visits for prenatal care during her pregnancy and prenatal care was considered insufficient if there were less than 3 visits for prenatal care during the course of the pregnancy. Birth weight was defined as the first measurement of body weight, usually in the first hour of life. Gestational age was calculated using the first day of last maternal menstrual period if it was known, or estimated by obstetric sonography, or with the Dubowitz score. Fetal macrosomia was defined as birth weight above the 90th percentile of the Leroy and Lefort curve. Prematurity was defined as a birth occurring before 37 completed weeks of gestation. Post-term was defined as 42 or more weeks of gestation. Birth asphyxia was defined as a low Apgar score of less than 7 at 5 minutes [7]. Respiratory morbidity was defined by the presence of tachypnea or chest retractions regardless of the etiology. Fresh stillbirth was defined as the intrauterine death of a fetus during labor or delivery. Early neonatal mortality included any death that occurred within the first 7 days of life. Perinatal mortality was defined as the sum of all stillbirths and early neonatal deaths.

**Statistical analysis:** Statistics such as percentage, mean, and standard deviation were used to describe the data. Pearson's chi-square test (for categorical variables) or Student's t-test (for continuous variables) were performed to determine the association between the various factors under investigation. A P-value of less than 0.05 was considered statistically significant.

## Results

**General data:** Overall, 588 cases of cesarean section were carried during the study period among a total of 3297 births. Cesarean section deliveries accounted for 17.83% of all births. There were a total of 142 (24.15%) elective cesarean sections which were compared to 446 (75.85%) emergency sections. Both groups were comparable in demographic, social, and past obstetric history characteristics. There were no differences in the experiences of surgeons compared to the operative techniques. Durations of surgery were also comparable between the two groups. Table 1 shows maternal, pregnancy, delivery, and newborn characteristics.

**Table 1:** Maternal, pregnancy, delivery, and newborn characteristics*

Characteristics	Elective cesarean (n = 142)	Emergency cesarean (n = 446)	*P*-value
**Maternal characteristics**			
Mean maternal age (years)	31.5 ± 6.54	27.8 ± 6.07	< 0.001
Maternal age greater than 40 years	3 (2.11)	11 (2.47)	0.810
Maternal age less than 18 years	1 (0.70)	4 (0.90)	0.828
Single woman	2 (1.41)	10 (2.24)	0.541
Rural residency	13 (9.15)	63 (14.13)	0.124
Maternal illiteracy	26 (18.31)	118 (26.46)	0.049
**Pregnancy characteristics**			
Primiparous mother	41 (28.87)	188 (42.15)	0.005
Maternal chronic disease	17 (11.97)	51 (11.43)	0.862
Multiple pregnancy	6 (4.23)	34 (7.62)	0.161
Insufficient prenatal care	14 (9.86)	179 (40.13)	< 0.001
Gestational diabetes mellitus	17 (11.97)	22 (4.93)	0.003
Gestational hypertension	10 (7.04)	14 (3.14)	0.041
Prelabor rupture of membranes greater than 18 hours	24 (16.90)	89 (19.96)	0.421
Referred from other institution for pregnancy complications or delivery	3 (2.11)	70 (15.70)	< 0.001
**Delivery characteristics**			
Breech or other malpresentation	16 (11.27)	41 (9.19)	0.467
Chorioamnionitis	0 (0)	8 (1.79)	0.108
General anesthesia during cesarean rather than regional block	0 (0)	38 (8.52)	< 0.001
**Newborn characteristics**			
Male	69 (48.59)	231 (51.79)	0.506
Female	73 (51.41)	215 (48.21)	0.506
Mean birth weight (grams)	3258 ± 614	3111 ± 687	0.016
Mean gestational age (weeks)	38.45 ± 2.65	38.53 ± 0.96	0.725
Prematurity	1 (0.70)	21 (4.71)	0.029
Birth asphyxia	3 (2.11)	29 (6.50)	0.045
Respiratory morbidity	5 (3.52)	43 (9.64)	0.020
Perinatal mortality	0 (0)	6 (1.35)	0.165
Admission in neonatal intensive care unit	7 (4.93)	51 (11.43)	0.024

* Values are given as mean ± standard deviation and number (percentage).

**Maternal data:** The youngest woman included in the study was 16 year old and the oldest was 46 years old. Elective cesarean sections were globally performed in older mothers with a mean age of 31.5 ± 6.54 years. On the other hand, emergency cesarean sections were performed in younger mothers with a mean age of 27.8 ± 6.07 years. This difference in the ages of mothers was statistically significant (P < 0.001). There were also statistically more primiparous mothers in emergency cesarean group than in elective cesarean group (P = 0.005), since 42.15% of mothers were primiparous in emergency cesarean group and only 28.87% were primiparous in elective cesarean group. Also, elective versus emergency cesarean was statistically associated with gestational diabetes mellitus (P = 0.003) and gestational hypertension (P = 0.041). Of mothers who received adequate prenatal care (67.18%), 67.59% underwent emergency cesarean section versus 92.75% among mothers who received insufficient prenatal care and there was a statistically significant relationship between emergency cesarean section versus elective cesarean regarding insufficient prenatal care (P < 0.001). Referred mothers accounted for 15.70% of emergency cesarean sections versus 2.11% of elective cesarean sections and the difference was statistically significant (P < 0.001). All mothers who underwent elective cesarean section were operated under regional anesthesia, while general anesthesia was given to 8.52% of mothers who underwent emergency cesarean section. Moreover, there was a statistically significant relationship between emergency cesarean section versus elective cesarean section regarding maternal illiteracy (P = 0.049). On the contrary, there was no significant difference in elective versus emergency cesarean groups in terms of marital status, area of residency, incidence of maternal chronic disease, multiple pregnancy, prelabor rupture of membranes greater than 18 hours, malpresentation, or chorioamnionitis.

**Fetal outcome:** Out of 588 newborns, 583 (99.15%) were born alive. Perinatal mortality in this study was 10.2 per 1000 births, consisting of 5 fresh stillbirths and 1 case of early neonatal mortality related to birth asphyxia. All these deaths were of the emergency cesarean group. Furthermore, there was statistically significant difference in prematurity (P = 0.029), birth asphyxia (P = 0.045), respiratory morbidity (P = 0.020) in emergency cesarean compared with elective cesarean sections. In elective cesarean group, 0.7% of the newborns were preterm and the remaining was term. In emergency cesarean group, 4.71% of the newborns were preterm, 8.74% were post-term and the remaining was term. Birth asphyxia was higher in emergency cesarean group (4.04%) as compared to elective cesarean group (2.11%). Respiratory morbidity was the most common fetal complication, seen in 48 cases (8.16%) of which 43 (89.58%) were from the emergency cesarean group. Mean gestational age in which cesarean section was done was similar in both groups, that is 38 and half weeks. Newborns in emergency cesarean group had lower birth weight (3258 ± 614 g) than in elective cesarean group (3111 ± 687 g) and the difference was statistically significant (P = 0.016). A soft tissue injury was encountered in one newborn of emergency cesarean group. Admission in neonatal intensive care unit was required in 9.86% of which 12.07% were in elective cesarean group and 87.93% were in emergency cesarean group. This difference was statistically significant (P = 0.024).

**Indications of cesarean sections:** The usual indications of elective cesarean sections were dominated by previous cesarean section (47.18%) and fetal macrosomia (17.61%). The most frequent indications for emergency cesarean section were fetal distress (30.49%) and previous cesarean section in labour (29.82%). The main indications for cesarean section in relation to the type of cesarean section are shown in Table 2. There was a statistically significant association between some of these indications and the type of cesarean section delivery. Other maternal indications were ischemic heart disease, glaucoma, genital herpes or extensive condyloma acuminate, and suspected or imminent uterine rupture.

**Table 2:** Cesarean section delivery indications*

Indications	Elective cesarean (n = 142)	Emergency cesarean (n = 446)	*P*-value
**Fetal indications**			
Fetal distress	0 (0)	136 (30.49)	< 0.001
Multiple pregnancy	18 (12.68)	31 (6.95)	0.032
Fetal macrosomia Severe intrauterine growth restriction	25 (17.61) 3 (2.11)	74 (16.59) 8 (1.79)	0.779 0.807
Post-term	0 (0)	39 (8.74)	< 0.001
Fetal hydrocephaly	5 (3.52)	3 (0.67)	0.011
Precious baby	3 (2.11)	11 (2.47)	0.810
**Extraembryonic membranes indications**			
Prelabor rupture of membranes greater than 48 hours	16 (11.27)	67 (15.02)	0.263
Chorioamnionitis	0 (0)	11 (2.47)	0.059
Placenta previa	5 (3.52)	14 (3.14)	0.823
Placental abruption	0 (0)	9 (2.02)	0.088
Cord prolapse	0 (0)	14 (3.14)	0.033
Severe oligohydramnios	0 (0)	8 (1.79)	0.108
**Dystocia indications**			
Cephalopelvic disproportion	13 (9.15)	64 (14.35)	0.110
Failure to induce labour	0 (0)	15 (3.36)	0.027
Non progress of labor	0 (0)	30 (6.73)	0.002
Breech presentation	9 (6.34)	47 (10.54)	0.138
Other malpresentation	5 (3.52)	28 (6.28)	0.214
**Maternal indications**			
Previous cesarean delivery	67 (47.18)	133 (29.82)	0.001
History of miscarriage, perinatal death, or infertility	8 (5.63)	5 (1.12)	0.001
Severe pre-eclampsia, eclampsia, or HELLP syndrome	0 (0)	36 (8.07)	0.017
Other maternal illness	1 (0.70)	7 (1.57)	0.438

* Values are given as number (percentage).

## Discussion

Cesarean sections have long been practiced as an obstetric surgical procedure that contributes to reducing fetal complications. And though it is classified as a major procedure, the incidence of cesarean section has considerably increased over the years all over the world [1]. Nevertheless, its advantages do not justify its continuous increase since it carries considerable disadvantages when compared with normal vaginal delivery. According to some studies, cesarean section requires a longer recovery time and operative complications such as lacerations and bleeding may occur at rates varying from 6% for elective cesarean to 15% for emergency cesarean [8, 9]. Though advances in the field have reduced maternal complications considerably, the problem of fetal morbidity after cesarean section still persists. And as much as is practical, everything points to the advantages that can be derived from an elective cesarean as compared to one that is undertaken as an emergency [10]. During the study period, the incidence of cesarean section at Souissi maternity hospital was found to be 17.83% and the overall cesarean section delivery rate was 24.15% for elective cesarean sections and 75.85% for emergency cesarean sections giving an approximate ratio of 4:1 for emergency versus elective cesarean section. Najam et al. and Ali et al. conducted two studies in India and Pakistan and reported a cesarean section rate of respectively 19.2% and 17.65% which is comparable to our results [11, 12]. The cesarean section rate found in our study was yet lower than the US rate of 32.2% in 2014 [13]. This rate does not however reflect true cesarean births in Morocco. Souissi maternity hospital being a tertiary referral centre for many health centers with limited resources and receives complicated cases of the catchment area. Similarly, many cesarean sections are done at private hospitals. A study done in Croatia in 2006 found 18% cesarean section rate out of which 48% were elective and 52% were emergency cesarean sections [14]. In another study conducted in Australia in 2005, the incidence of cesarean section was 28.3% of which 35.8% were elective and 64.14% were cesarean emergency sections [15]. In Nigeria, Onankpa et al. reported a cesarean section rate of 8.4%. Of these, 19.4% were elective and 80.6% were emergency cesarean sections. As stated by the authors, cesarean section deliveries are not readily accepted by the mothers in their country which explains such low rate of cesarean section deliveries [16]. As previously reported by Al Nuiam et al., significant difference was found between emergency cesarean delivery and younger mothers and low parity in this study [17]. The relationship of age with the type of cesarean section is difficult to decipher. However, the high incidence of emergency cesarean section in younger mothers may indicate the tendency of the attending obstetrician to allow vaginal deliveries in these mothers as long as this is feasible with a view to preserving their future reproductive performances and only resorting to cesarean section delivery when there is a threat to either the mother or the fetus. On the other hand, it is accepted that the older mothers tend to have more previous cesarean section deliveries, which may automatically require elective cesarean section. In this study, cesarean section delivery was performed on primiparous mothers in 38.95% of cases. Other studies found a slightly higher rate. It was 42% for Kambo et al. and 55.48% for Adhikeri et al. [18, 19].

Overall, fetal complications were higher in emergency cesarean group. Fetal morbidity was 28.23%. Of this, 90.36% cases were contributed by the emergency cesarean group and 9.64% were elective cesarean group. The major cause of fetal morbidity was respiratory morbidity followed by birth asphyxia, seen mainly in emergency group. Prematurity, birth asphyxia, respiratory morbidity, and admission in neonatal intensive care unit were significantly more frequent in emergency cesarean group than in elective cesarean group. Other studies have reported similar facts [11, 14, 16]. De Luca et al. found in their study that there was less fetal morbidity in elective cesarean group than in emergency cesarean group section but perinatal mortality and respiratory morbidity were similar in both groups [20]. This was contrary to the findings of Miller et al. [21]. They reported in their study that birth asphyxia was less common in emergency cesarean section than in elective cesarean section. This is difficult to explain except for the fact that in their study emergency cesarean section was most often carried out to save the fetus. Besides, transient tachypnea of the newborn may follow cesarean section, especially if it is elective cesarean section. A debate exists as to whether cesarean section delivery contributes to the genesis of this disease. Kamath et al. compared elective repeat cesarean delivery and vaginal birth after cesarean and concluded that neonates born after elective repeat cesarean delivery have significantly higher rates of respiratory morbidity and admission in neonatal intensive care unit [22]. However, Lopez et al. found opposite results in their study [23]. Roth-Kleiner et al. found that severity of respiratory morbidity was higher in newborns after elective cesarean section than in emergency cesarean section, probably because of the changes occurring to the fetal lungs when the mother gets into labor [24]. Those findings do not correlate with ours though. Moreover, elective repeat cesarean section has been implicated in the development of pulmonary hypertension of the newborn [25]. Furthermore, a common cause of fetal complications is infant respiratory distress syndrome which is a function of gestational age [26]. Inappropriately timed cesarean delivery has been known to result in this complication. According to a study by Morrison et al., a significant reduction in neonatal respiratory morbidity can be obtained if elective cesarean section is performed during the 39th week of pregnancy [27]. Perinatal mortality was 10.2 per 1000 births and was only observed in emergency cesarean group. There was one early neonatal death in this group due to hypoxic encephalopathy, as also found in Cebeku et al. study [28]. This was in spite of the fact that all antenatal complications that might predispose to adverse fetal outcomes were excluded from the study. Studies from developed countries have reported a perinatal mortality for cesarean section deliveries of less than 10 per 1000 births [14]. In developing countries, Onankpa et al. reported that perinatal mortality was 11 per 1000 among the cesarean deliveries [16]. Ali et al. reported a perinatal mortality for cesarean section deliveries of 10 per 1000 which was similar to our findings [12]. In both these studies the perinatal mortality was higher in emergency cesarean group.

In our study, the most frequent reason for cesarean section was a previous cesarean delivery (35.84%) which is similar as in literature [14, 15, 29]. The second most frequent indication of cesarean section in this study was fetal distress and it only concerned emergency cesarean sections. The most frequent indications of elective cesarean section were previous cesarean section delivery and fetal macrosomia. The most frequent indications for the emergency cesarean section were fetal distress and previous cesarean section in labour. In Elvedi-Gasparovic et al. study, the commonest indication of elective cesarean section was previous cesarean section whereas the commonest indication of emergency cesarean section was pre-eclampsia and eclampsia [14]. In Najam et al. study, the common indications were the same in elective cesarean group. But in emergency cesarean group, repeat cesarean section was the commonest indication followed by non progress of labor, eclampsia, pre-eclampsia, and cephalopelvic disproportion [11]. Ali et al. have reported in their study that in 43.24% cases, the indication for cesarean section was a previous cesarean delivery and malpresentation was the indication in 11.9% of cases [12]. One of the goals of prenatal care is to reduce pregnancy complications which may warrant emergency cesarean section. The finding of a significantly greater incidence of emergency cesarean section in mothers with insufficient prenatal care (40.13%), as compared with only 9.86% elective cesarean section, is in consonance with this concept. In the same manner, the correlation between most of the indications and the incidence of emergency cesarean section is not surprising, especially since most of these indications are the same factors that warrant emergency cesarean section in the first instance. Factors that contribute to the indications for emergency cesarean section like fetal distress, cephalopelvic disproportion, failure to induce labour, non progress of labor, and previous cesarean delivery have to be evaluated independently in a further study to assess the contribution of each factor to the fetal morbidity and mortality and how best these can be avoided. The duration of this study was a limitation. We however managed to know the current fetal outcome and rates of cesarean section at Souissi maternity hospital. This study will help to compare the current results with future trend later on.

## Conclusion

Emergency cesarean sections showed significantly more fetal complications than elective cesarean sections in this study. The high incidence of emergency cesarean section found emerges from insufficient prenatal care and poor referral system. Early recognition and referral of mothers who are likely to undergo cesarean section may reduce the incidence of emergency cesarean sections and thus decrease fetal complications.

### What is known about this topic

- Fetal complications are more commonly seen in emergency than in elective cesarean sections;
- To our knowledge, no previous study has evaluated fetal outcome in emergency versus elective cesarean sections in Morocco.

### What this study adds

- Emergency cesarean sections showed significantly more fetal complications than elective cesarean sections in this study;
- Incidence of emergency cesarean was high in this study due to insufficient prenatal care and poor referral system;
- Early recognition and referral of mothers who are likely to undergo cesarean section may reduce the incidence of emergency cesarean sections and thus decrease fetal complications.
